# Slow-exploring captive red knots were quicker to find food in a social setting than fast explorers

**DOI:** 10.1098/rsbl.2024.0139

**Published:** 2024-07-24

**Authors:** Aileen Roncoroni, Selin Ersoy, Allert I. Bijleveld

**Affiliations:** ^1^ Department of Coastal Systems, NIOZ Royal Netherlands Institute for Sea Research, Texel, The Netherlands; ^2^ Department of Earth Sciences, Utrecht University, Utrecht, The Netherlands; ^3^ Groningen Institute for Evolutionary Life Sciences (GELIFES), University of Groningen, Groningen, The Netherlands

**Keywords:** red knots, exploration, animal personality, foraging success, social information, producer–scrounger tactics

## Abstract

Individuals foraging in groups face increased competition but can benefit from social information on foraging opportunities that can ultimately increase survival. Personality traits can be associated with food-finding strategies, such as shyer individuals scrounging on the food discoveries of others. How personality and foraging strategy interact in a social foraging context with different group compositions received less attention. Here, we conducted experiments to investigate the relationship between exploratory personality, group size (1–4 birds) and foraging success (i.e. speed of finding a food patch) in wild-caught red knots. We found that faster explorers, when foraging alone, discover food patches quicker than slower explorers. In groups, however, slower-exploring birds became quicker at finding food than fast explorers. This shows that slower-exploring individuals benefit from group foraging. They seem to be more perceptive to social cues, and in contrast to faster explorers, they become quicker at finding food when they are in a group than when foraging alone. We discuss how individuals with different personalities and foraging strategies can coexist in a social foraging context with different costs and benefits associated with their strategies.

## 1. Introduction

Resources in natural systems are often unevenly distributed [1], so information on food patch location is crucial in finding food and, consequently, survival in the wild [2]. Individuals that live in groups (e.g. flocks of birds) face competition for food [3] but can also benefit from social information to locate food [4,5]. The Local Enhancement Hypothesis predicts that individuals are likely to be attracted to areas where conspecifics are feeding [6,7]. While some individuals are consistently finding new food locations independently, others scrounge on their counterparts’ discoveries (‘producer–scrounger tactics’ [8]). This phenomenon has been observed in a wide range of taxa [9,10]; however, research on the extent of individual differences in discovering food location in group foraging animals has been relatively limited (e.g. [11–13]).

Consistent among-individual differences in behaviour (known as animal personalities [14]) such as exploration (magnitude of space use in the novel environment) and boldness (likelihood of risk-taking) could be one of the factors predicting social foraging information use and food-finding in the wild (e.g. [15]). For example, faster-exploring great tits *Parus major* are more likely to discover new feeders than slower explorers [16], and shyer barnacle geese *Branta leucopsis* scrounge on the food discoveries of bolder geese [11]. Additionally, exploratory behaviour in zebra finches *Taeniopygia guttata* affects feeding success differently across social contexts [12]. Faster-exploring or bolder individuals may be more inclined to find food successfully themselves, which can attract slower-exploring or shyer individuals, thereby increasing competition. However, foraging in groups can still be beneficial as, for instance, it reduces predation risk [17]. Producer and scrounger food-finding tactics likely involve differences in costs and benefits (e.g. [18]). Because in the wild it is difficult to detect foraging information transfer, controlled captive studies are necessary.

Red knots *Calidris canutus* are shorebirds that forage in large groups on patchily distributed shellfish [19]. In laboratory experiments, red knots have been shown to differ in exploratory behaviour [20], use social information to detect the foraging success of conspecifics [21] and differ consistently in producer–scrounger tactics [21]. As personality has been shown to link with foraging tactics in other species (e.g. [11–13]), we may expect it to be the case in red knots also. Such investigation contributes to the growing body of literature aimed at understanding how personality interacts with group dynamics to influence foraging strategies and how consistent individual differences may be evolutionarily maintained.

In this study with wild-caught red knots brought into captivity, we investigated how food patch discovery (i.e. speed of finding hidden food) is affected by (i) exploratory personality, (ii) group size, and (iii) how these two variables interact. We first assessed the exploration speed of red knots under controlled conditions (exploratory personality assays). We then created an experimental foraging arena where food was patchily distributed and hidden such that individuals needed to search trays to find the one containing food. There, we tested each individual alone and in groups of 2–4 birds (foraging experiments). We predicted that faster-exploring red knots would discover the food patch quicker than slower explorers when foraging alone (versus in a group). In groups, slower explorers are expected to scrounge on the food discoveries made by other individuals; therefore, we predicted slower explorers would become quicker at finding the food patch when they are in a group (versus alone). Additionally, we predicted all individuals to become quicker at finding food with increasing group size because they are expected to use social information on the location of the food patch through local enhancement.

## 2. Methods

### (a) Study species

Fifty red knots of the *islandica* subspecies were captured with mist-nets in the Dutch Wadden Sea near the island of Griend (53°15ʹ N, 5°15ʹ E) in October 2019 and transported to the NIOZ Royal Netherlands Institute for Sea Research (53°00ʹ12.1ʺ N, 4°47ʹ23.3ʺ E). Each bird was provided with leg rings for individual identification. The birds were housed in outdoor aviaries (approx. 4 m × 2 m × 2.5 m) equipped with running freshwater in a tray, a stretch of sand covered in 5 cm saltwater and running saltwater on the floors. The aviary composition was randomly changed daily to prevent dominance-related attacks, ensuring optimal feeding conditions. The birds were fed with protein-rich trout food pellets (Produits Trouw, Vervins, France).

### (b) Exploratory personality assays

After three months of acclimatization to captivity, exploratory personality was assessed in a novel environment (identical to [20] and [22]). The experimental arena (7 m × 7 m × 3 m) was filled with 30 cm seawater and food-free wet sand patches (1 m × 1 m × 35 cm). We recorded and tracked the movement of the bird (using idTracker software [23]). We computed position data (i.e. *x* and *y* coordinates) every 0.5 s during the entire time a bird spent in the arena (20 min) and calculated the movement speed. The log of the mean speed (metres per second) was then used as a measure for exploration speed. For more information about personality assays, see Bijleveld *et al*. [20] and Ersoy *et al.* [24].

### (c) Foraging experiments

**Figure 1.**
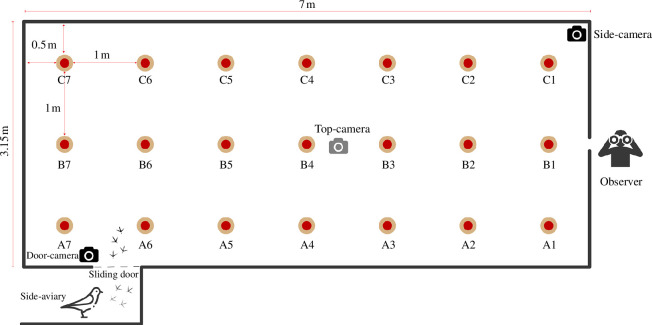
Illustration showing the experimental arena with the location and labels of the patches of which only one randomly assigned had food in it.

Foraging experiments were conducted in an arena filled with sand (approx. 7 m × 3.15 m × 3 m). The adjacent side-aviary (4 m × 1.6 m × 2.5 m) was used to acclimatize the birds before the trial and to release them into the experimental arena through a sliding door. Twenty-one trays were placed in the experimental arena (figure 1) with only one containing hidden food, hereafter called the ‘food patch’ (electronic supplementary material, figures S1 and S2), whose location was randomly selected for each trial or group of trials with different individuals. Three GoPro cameras filmed the trials from different angles: (i) top-camera on the ceiling; (ii) door-camera in the side-aviary; and (iii) side-camera on the side of the arena, near the observer (electronic supplementary material, videos S1–S3).

To familiarize birds with the set-up and teach them that only one patch contained food, we conducted a four week training period in which we repeatedly introduced the birds into the arena. We initially released large groups of 25 individuals overnight, and then we progressively reduced the group size and time interval until the same conditions of the experimental trials were met. Furthermore, we gradually decreased the number of filled food patches in the arena from 6 to 1, and the location was randomly selected each time.

After training, we ran a total of 200 trials (50 birds × 4 trials of different group sizes). We first tested the birds individually in the arena (*N*_ind_ = 50) and then with other randomly selected individuals forming groups of two (*N*_2_ = 50), three (*N*_3_ = 50) and four (*N*_4_ = 50). In group experiments, we analysed only the focal bird’s behaviour (i.e. not the partner’s behavior). To eliminate the effects of time of day and the sequence in which the trials took place, we randomly assigned the order of trials between and within days. To motivate birds to search for food during the experiments, we deprived them of food from the night before (12–18 h). Each trial consisted of retrieving the focal bird (and the partners in group experiments) and letting it acclimate in the side-aviary for an average of 8.2 min (s.d. = 2.4 min). Then, we remotely opened the sliding door connecting the side-aviary to the arena with a pulley mechanism to allow entry (figure 1). If the birds did not enter voluntarily after 2 min, we gently herded them inside. Regardless of how long it took for the focal bird to fly into the arena, we measured the time from opening the door to the discovery of the food patch. We defined a food patch as ‘discovered’ when the focal bird reached the food patch with its bill and began eating from it. Trials were ended 2 min after the food was found, allowing a reward to the bird. In case the focal bird did not search for or did not discover the food patch (in some instances, the food patch was monopolized by an individual, hindering others’ access to food), we ended trials after 10 min. A video of an experiment is provided as an example in the electronic supplementary material, videos S1–S3.

### (d) Statistical analyses

Data analyses were carried out in R v. 4.0.3 [25]. We formulated a linear mixed-effect model using the *lme4* package after checking for collinearity, overdispersion and model assumptions (homogeneity and normality of residuals) [26]. The response variable was the time taken to find the food patch in seconds and it was log-transformed to meet normality assumptions. We added exploration speed (log cm s^−1^), group size (1–4) and the interaction between the two as fixed effects. To control for repeated measures on individuals, we added the focal bird ID nested in group size and the location ID of the food patch as random effects. The effects were assessed based on whether the confidence interval crossed zero.

## 3. Results

**Figure 2.**
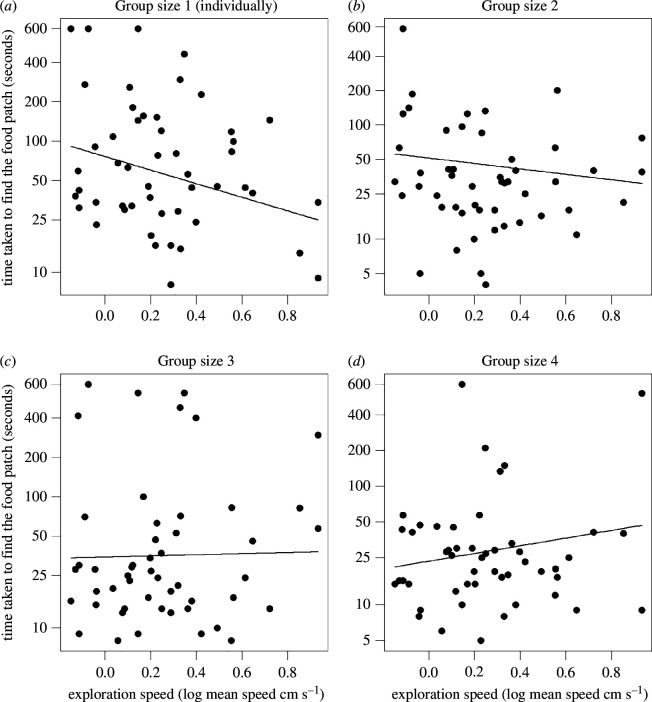
Exploration speed (log cm s^−1^) interacting with group size (1–4) predicts the time it takes a bird to locate the food patch (seconds). Each plot shows the group size at which the focal bird was tested. Individually (*a*), faster-exploring red knots located the food patch faster than slower-exploring birds. With increasing group size (2, 3 and 4, respectively, panels *b*,*c* and *d*), this difference reversed, and with groups of four birds, the slower explorers found the food patch quicker than the faster explorers.

**Figure 3.**
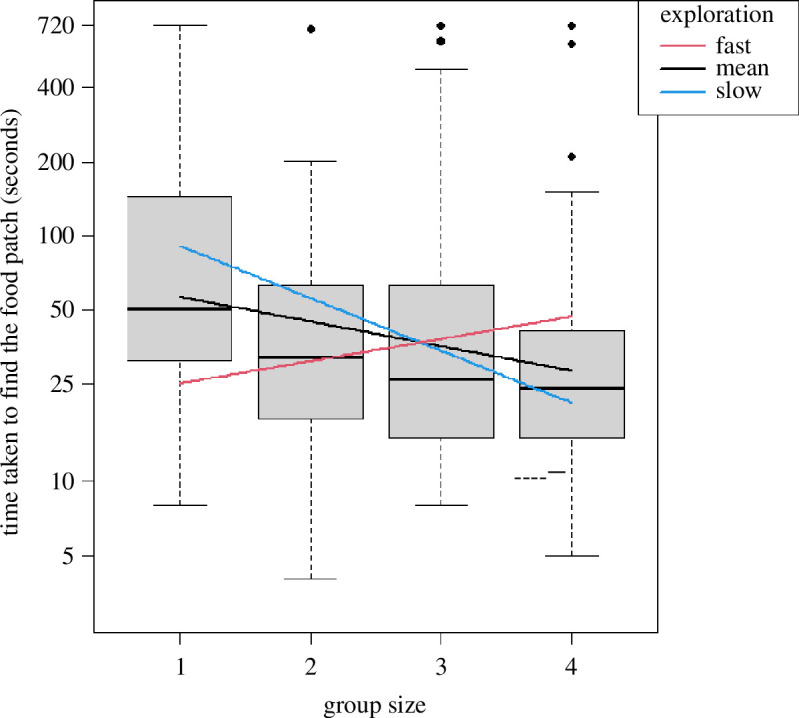
The time it took a bird to locate the food patch (seconds) in relation to group size and exploration speed (log cm s^−1^). On average, all individuals became quicker in finding the food patch with increasing group size; however, faster-exploring individuals became slower. For visualization purposes, predictions are presented as lines for grouped exploration behaviour: 20 individuals with the highest exploration speeds are labelled as ‘fast’, and the 20 birds with the lowest exploration speeds are grouped as ‘slow’. The 10 birds with intermediate exploration speeds are the ‘mean’ exploration group.

We found that, when tested individually, faster-exploring individuals were quicker at locating the food patch than slower explorers (figure 2; table 1). The time to locate the food patch decreased with increasing group size (figure 3; table 1), indicating that on average all individuals became faster at food-finding when in a group. We also found a significant interaction effect between group size and exploration speed (table 1). With increasing group size, slower-exploring red knots became quicker at finding the food patch than faster-exploring red knots. In contrast, faster-exploring birds took increasingly longer to discover the food patch in groups compared to when foraging alone (figure 3; table 1).

**Table 1.** Results from the linear mixed-effect model investigating what factors correlated with the time to find the food patch. The bold values indicate that 95% confidence intervals do not overlap zero. ICC, intraclass correlation coefficient; *N*, sample size.

	time to find the food patch (seconds)	
predictors	estimates	95% CI
intercept	2.05	**1.82 to 2.29**
exploration speed (log mean speed cm s^−1^)	−0.8	**−1.37 to −0.23**
group size (1–4)	−0.17	**−0.25 to −0.09**
exploration speed: group size	0.28	**0.07 to 0.49**
random effects		
residual	0.15	
among-bird	0.08	
among-food location	0.03	
bird nested in group size	0.01	
ICC	0.39	
*N*_BirdID_	50	
*N*_Food location_	14	
observations	200	
marginal *R*^2^/conditional *R*^2^	0.079 /0.436	

## 4. Discussion

In this study, we investigated how foraging success was influenced by personality and the presence of other individuals. We found that faster-exploring red knots locate food patches quicker than slower explorers when tested individually. However, slower explorers became quicker at finding food than faster-exploring birds when foraging in groups. Also, compared to foraging alone, faster explorers became slower when foraging in groups. Our findings show that slower-exploring individuals benefit from group foraging in contrast to faster-exploring individuals.

Group-living individuals can differ in their foraging strategies, as some individuals are consistently better at discovering new food locations than their conspecifics [8]. Individuals with a fast exploratory personality are shown to be faster at locating resources than slow explorers [16]. Indeed, we also found that faster-exploring red knots are quicker at finding food when foraging alone than slower explorers. Slower-exploring individuals, however, became quicker at locating food in the presence of other individuals compared to faster explorers. These results could suggest that slower explorers are likely to be the scroungers on the information produced by others in a group. However, since our study did not provide direct evidence that slow explorers followed fast explorers to find food sources, it is plausible that slow explorers may have independently located food patches. This indicates a social effect that extends beyond information use, such as social facilitation [27]. The differences in foraging success depending on the social context might be related to slower-exploring birds being more flexible in responding to cues in the (social) environment compared to faster-exploring individuals who are less responsive to such clues [28,29]. Indeed, previous studies highlight that slow individuals are more reactive and change their behaviour according to environmental cues than fast individuals, who are more prone to routine behaviours (e.g. [30]).

In their natural habitat in the Wadden Sea, red knots mainly forage on patchily distributed shellfish buried in mudflats, thus obscured from sight [19]. Such foraging habitat provides potential for local enhancement [6] as using social foraging information likely increases the chances of finding hidden food and ultimately the foraging success of the individual. A recent study, combining personality assays and high-throughput tracking, revealed that exploratory personality correlates with movement in the wild, and faster-exploring red knots visit more areas than slower explorers [31]. Thereby, faster explorers can find new foraging areas and provide foraging information to their conspecifics. Whether faster explorers are the producers that are followed by slower explorers should be studied further, using high-resolution tracking combined with personality assays, to be able to understand the actual role of personalities in the use of foraging information [32]. Mudflats additionally present time constraints as the availability of foraging habitat is driven by the tide [19], and finding food within limited periods when mudflats are accessible poses potential survival consequences. Our experimental set-up with limited food patches showed very short time differences in finding food among individuals (not more than 1 min); however, these time differences observed in controlled settings are likely to be amplified in the field where birds forage on a larger scale with scattered food sources. While more research is needed to understand the individual composition of foraging red knot groups in the wild, our experimental study with birds that were brought from wild to captivity for a limited time frame is a step forward in understanding how individual personality is associated with social foraging strategies and group size.

Individuals with different personalities may experience different costs and benefits associated with their foraging strategies that overall could provide equal fitness. For example, faster individuals are expected to have costs associated with finding food patches, such as flight costs, and their discoveries attract slower explorers, which increase competition for food. In particular, as our results showed, fast explorers become slower at finding food in groups, meaning there is a negative effect of exploration speed on food-finding time within a social context. However, the costs of both producing and being scrounged upon could be offset by different benefits that might explain the variation in behaviour among individuals. Firstly, being in a group reduces predation risk [17], and while faster explorers might have poor attention to the environment, slower explorers can react readily to changes [28]. Indeed, fast explorers may profit from the presence of slow explorers because of their increased vigilance [33], and *vice versa*, slow explorers may profit from fast explorers because of their food-finding ability. Secondly, modelling studies have shown that among-individual variation may be maintained when producers have a ‘finders-fee’, meaning they gain an advantage over scroungers by consuming a greater quantity of prey in the discovered food patch [8]. Thirdly, as food patches on the mudflats are large, the costs arising from competition are small because birds can spread out to avoid physical encounters with conspecifics [34]. How these costs and benefits of different foraging strategies affect fitness and how they offset each other to potentially maintain between-individual variation within populations remain to be studied [35].

## Data Availability

Data and code supporting this manuscript can be accessed through the electronic supplementary material [36].
